# Enhancement Characteristics and Impact on Image Quality of Two Gadolinium Chelates at Equimolar Doses for Time-Resolved 3-Tesla MR-Angiography of the Calf Station

**DOI:** 10.1371/journal.pone.0099079

**Published:** 2014-06-03

**Authors:** Jan Hansmann, Henrik J. Michaely, John N. Morelli, André Luckscheiter, Stefan O. Schoenberg, Ulrike I. Attenberger

**Affiliations:** 1 Institute of Clinical Radiology and Nuclear Medicine, University Medical Center Mannheim, Medical Faculty Mannheim – Heidelberg University, Mannheim, Germany; 2 The Russel H. Morgan Department of Radiology and Radiological Science, The Johns Hopkins Hospital, Baltimore, Maryland, United States of America; West German Cancer Center, Germany

## Abstract

**Purpose:**

To compare enhancement characteristics and image quality of two macrocyclic gadolinium chelates, gadoterate meglumine and gadobutrol, in low-dose, time-resolved MRA of the calf station.

**Materials and Methods:**

100 consecutive patients with peripheral arterial disease (stages II-IV) were retrospectively analysed. Fifty patients were included in each group - 32 men and 18 women for gadobutrol (mean age 67 years) and 34 men, 16 women for gadoterate meglumine (mean age 64 years). 0.03 mmol/kg bw of either gadobutrol or gadoterate meglumine was injected. Gadobutrol was diluted 1∶1 with normal saline (0.9% NaCl) to provide similar injection volume and bolus geometry compared to the undiluted 0.5 M dose of gadoterate meglumine. Signal-to-noise-ratio (SNR), contrast-to-noise-ratio (CNR) and image quality were analysed and compared between the two groups.

**Results:**

Mean SNR ranged from 83.0±46.7 (peroneal artery) to 96.4±64.5 (anterior tibial artery) for gadobutrol, and from 37.6±13.8 (peroneal artery) to 45.3±16.4 (anterior tibial artery) for the gadoterate meglumine group (p<0.0001). CNR values ranged from 30.1±20.1 (peroneal artery) to 37.6±26.0 (anterior tibial artery) for gadobutrol and from 14.9±8.0 (peroneal artery) to 18.6±16.4 (anterior tibial artery) for gadoterate meglumine (p<0.0001). No significant difference in image quality was found except for the peroneal arteries (p = 0.006 and p = 0.04). Interreader agreement was excellent (kappa 0.87–0.93)

**Conclusion:**

The significantly better enhancement as assessed by SNR and CNR provided by gadobutrol compared to gadoterate meglumine does not translate into substantial differences in image quality in an equimolar, low-dose, time-resolved MRA protocol of the calves.

## Introduction

The diagnostic value of contrast-enhanced MR-angiography (CE MRA) for the evaluation of the lower extremity vasculature at 3 Tesla (3 T) is well-established in the literature [1], [2]. Increasingly available high field MR systems operating at 3 T allow markedly shortened acquisition times without a corresponding loss in spatial resolution [1]. This is in part due to intrinsically higher SNR at these field strengths combined with the development of other innovations such as parallel imaging and multi-element coils [3],[4]. In particular, CE MRA enables accurate gradation of disease stage in patients suffering from peripheral artery disease (PAD): comparisons with conventional angiography, the clinical gold standard, have demonstrated a high degree of overall diagnostic accuracy with CE MRA, with values of 80% for stenosis detection and 93.5% for the detection of high grade vessel stenosis [5].

Evaluation of the relatively small caliber vasculature of the calf station is often impaired by venous overlay due to altered patient hemodynamics. This may result from soft tissue inflammation—a common finding in patients with advanced stages of PAD. Such limitations can be successfully avoided through implementation of time-resolved MRA sequences such as time-resolved imaging of contrast kinetics (TRICKS) [6] and time-resolved angiography with interleaved stochastic trajectories (TWIST). These sequences eliminate the need for a test-bolus and have been shown to provide arterial-phase imaging free of venous contamination in different vascular territories [7]. In peripheral MR-angiography applications, time-resolved MRA sequences have been proven to allow for an accurate assessment even in patients whose CE MRA images had been non-diagnostic due to venous contamination [8], [9].

Patients suffering from PAD also often present with multiple comorbidities including impaired renal function and are thus at an increased risk of developing nephrogenic systemic fibrosis (NSF). The incidence of NSF is not only related to the molecular structure of the administered gadolinium chelate, with macrocyclic chelates being more stable than linear compounds, but also to the injected dose. Therefore, low-dose injection protocols or nonenhanced MRA [10], [11] are preferred to minimize the risk of NSF in this particular patient group [4], [5]. The implementation of low-dose protocols together with the increased use of macrocyclic MR contrast agents has led to a significantly reduced risk for NSF in high-risk patients [12], [13]. Among the macrocyclic chelates available, gadobutrol is unique in its availability at an 1.0 M formulation and its higher r^1^ relaxivity when compared to other contrast agents. We hypothesized that the latter factor should thus lead to improvements in signal to noise ratio (SNR), contrast to noise ratio (CNR) and image quality [14], [15].

The aim of the current investigation was therefore to compare equimolar doses of gadobutrol and gadoterate meglumine with respect to SNR, CNR and image quality in a time-resolved, low-dose MRA protocol of the calf region.

## Materials and Methods

### Patients

Institutional review board approval was obtained for this study. The institutional review board (IRB name: Medizinische Ethikkommission II der Medizinischen Fakultaet Mannheim, Heidelberg University, Germany) waived the requirement for informed patient consent for this retrospective study. The information gathered on this retrospective patient population was performed in compliance with HIPAA guidelines. 100 consecutive patients with PAD (Fontaine stages II-IV) who underwent routine MRA of the peripheral vasculature at our institution between October 2008 and March 2011 were included for retrospective data analysis (32 men and 18 women; mean age 67 years in gadubutrol group and 34 men, 16 women for gadoterate meglumine, mean age 64 years in the gadoterate meglumine group). Either gadobutrol or gadoterate meglumine was utilized as the contrast agent, based upon the clinical necessities of the examination day (i.e. patient renal function, contrast agent utilized for previous examinations).

### MR-Protocol

All MRA examinations were performed on a 3 T, 32-channel whole-body MR system (MAGNETOM Tim Trio [102×32], Siemens AG, Healthcare Sector, Erlangen, Germany). To cover the entire field of view (FoV) from the diaphragm to the calves, a dedicated 36-element peripheral angiography matrix coil, as well as 2 body 6-element matrix coils and 2 clusters of the inbuilt spine matrix coil were utilized. Patients were positioned supine feet-first in the MR bore. A 20 G cannula in the left or right antecubital vein was used for contrast agent administration, which was performed with an automated power injector (Medrad Spectris Solaris EP, Medrad Indianola, PA). Detailed scan parameters are summarized in Table 1.

**Table 1:** Sequence parameters for TWIST MR Angiography.

Parameter	TWIST MRA
Parallel Imaging	Grappa 2
Acquisition time (sec)	96
Spatial resolution (mm^3^)	1.1×1.1×1.1
Temporal resolution (sec)	5.49
Field of view (mm)	500×375
Repetition time (msec)	3.75
Echo time (msec)	1.12
Flip angle (degrees)	20
Matrix	448×336
Bandwidth (Hertz per pixel)	660

### Contrast agents

Two macrocyclic gadolinium based contrast agents were utilized for this study: gadobutrol (Gadovist, Bayer Healthcare AG, Berlin, Germany) formulated at 1.0 molar (M) and gadoterate meglumine (Dotarem, Guerbet, France) formulated at 0.5 M. The r^1^ relaxivity of gadobutrol versus gadoterate meglumine is 5.0±0.3 vs. 3.5±0.2 L*mmol^−1^s^−1^ (in plasma, at 3 T and 37°C) [15]. To allow for a more equal comparison between the two different contrast agents in terms of bolus geometry the 1 M gadobutrol was diluted 1∶1 with normal saline (0.9% NaCl). Thus, contrast agents administered to the patients were equimolar on a per bodyweight basis (0.03 mmol/kg BW) and were administered in equivalent concentrations (0.5 M).

### MR imaging

2D gradient echo sequence localizers were obtained in coronal and axial planes to allow for planning of the examination. After acquisition of continuous table movement-MRA (CTM-MRA) images, an additional 0.03 mmol/kg body weight of the respective contrast agent was administered at a rate of 1.5 ml/s followed by a 30 ml normal saline (0.9% NaCl) chaser at the same injection rate for time-resolved MRA with interleaved stochastic trajectories (TWIST) of the calf station.

### SNR/CNR evaluation

Image evaluation was performed offline utilizing a standard DICOM-viewer (OsiriX, The OsiriX Foundation, Geneva, Switzerland). Region-of-Interest (ROI) measurements were performed by a single reader for SNR and CNR measurements. The reader was blinded to the contrast agent administered. ROIs were placed in vessel segments in the anterior and posterior tibial as well as peroneal arteries on the pre-contrast scans. Noise calculations were performed by placing a ROI in an artifact-free background area of the subtracted coronal maximum intensity projection images. A ROI was placed in the corresponding vessel at consecutive time points and signal intensity then was measured at the time point of maximum vessel enhancement. The above ROIs were carefully placed in vessel segments displaying homogenous contrast enhancement while avoiding the vessel borders. SNR and CNR were calculated according to the following formulas [16]: 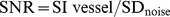
(1)

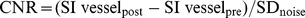
(2)


### Qualitative assessment

For the qualitative assessment, two blinded readers assessed image quality on a scale of 1 to 4 (1 =  poor image quality and blurring of the arterial segment; 2 =  fair image quality, inadequate arterial enhancement for confident diagnosis; 3 =  good image quality and arterial enhancement, adequate for confident diagnosis; 4 =  excellent image quality and arterial enhancement, for highly confident diagnosis) within 4 arterial segments in the bilateral lower extremities (tibioperoneal trunk, anterior tibial, posterior tibial, and the peroneal arteries). Data from both limbs in the patients was pooled, resulting in possible evaluation of 96 (2×48) data points for each territory. Territories in a given patient were excluded if arterial occlusions within a segment led to no significant vascular enhancement, one of the limbs was amputated, or vascular evaluation was not possible secondary to patient motion.

### Statistical analysis

Statistical analysis was performed using JMP 9.0 (SAS Institute, Cary, North Carolina, USA). Continuous variables are expressed as mean, standard deviation (± SD), and range (min–max). Continuous variables for SNR and CNR are expressed as mean and standard deviation. SNR and CNR values between the two groups were compared using a Student t-test after normally distribution of data was confirmed using the Shapiro–Wilk test. A 2-tailed p-value of <0.05 was considered statistically significant. To assess qualitative differences in quality between scans performed with gadobutrol and gadoterate meglumine, median image quality for each reader in 4 arterial segments was computed. Image quality ratings for each reader were compared between images obtained with gadobutrol and gadoterate meglumine utilizing a Mann-Whitney U Test. Inter-reader variability was assessed via Cohen's Kappa statistics. Kappa values greater than 0.75 were taken to represent excellent agreement, values between 0.4-0.75 to represent good agreement, and values below 0.4 as poor agreement.

## Results

### SNR/CNR evaluation

All measurements were completed successfully, and no examinations were excluded due to inadequate image quality. SNR measurements for gadobutrol demonstrated a mean signal intensity of 83.0±46.7 for the peroneal artery, 92.9±58.9 for the posterior tibial artery, and 96.4±64.5 for the anterior tibial artery. Gadoterate meglumine SNR was 37.6±13.8 for the peroneal artery, 41.2±13.8 for the posterior tibial artery, and 45.3±16.4 for the anterior tibial artery. SNR measurements were statistically significantly different for all three vessel segments evaluated (peroneal artery p<0.0001; posterior tibial artery p<0.0001, anterior tibial artery p<0.0001) (Table 2).

**Table 2:** SNR/CNR.

Contrast agent		Anterior tibial artery	Posterior tibial artery	Peroneal artery
**Gadobutrol**	*Mean SNR*	96.4±64.5	92.9±58.9	83.0±46.7
**Gadoterate meglumine**	*Mean SNR*	45.3±16.4	41.2±13.8	37.6±13.8
	*p-value*	*<0.0001*	*<0.0001*	*<0.0001*
**Gadobutrol**	*Mean CNR*	37.6±26.0	37.1±26.8	30.1±20.1
**Gadoterate meglumine**	*Mean CNR*	18.6±16.4	16.9±9.2	14.9±8.0
	*p-value*	*<0.0001*	*<0.0001*	*<0.0001*

Note – SNR  =  Signal to noise ratio; CNR  =  Contrast to noise ratio.

CNR measurements for gadobutrol in the peroneal artery demonstrated a mean signal intensity of 30.1±20.1 for the peroneal artery, 37.1±26.8 for the posterior tibial artery, and 37.6±26.0 for the anterior tibial artery. Gadoterate meglumine exhibited CNR of 14.9±8.0 for the peroneal artery, 16.9±9.2 for the posterior tibial artery and 18.6±16.4 for the anterior tibial artery. CNR measurements were statistically significantly different for all three vessel segments evaluated (peroneal artery p<0.0001; posterior tibial artery p<0.0001, anterior tibial artery p<0.0001) (Table 2).

### Qualitative assessment

**Figure 1:**
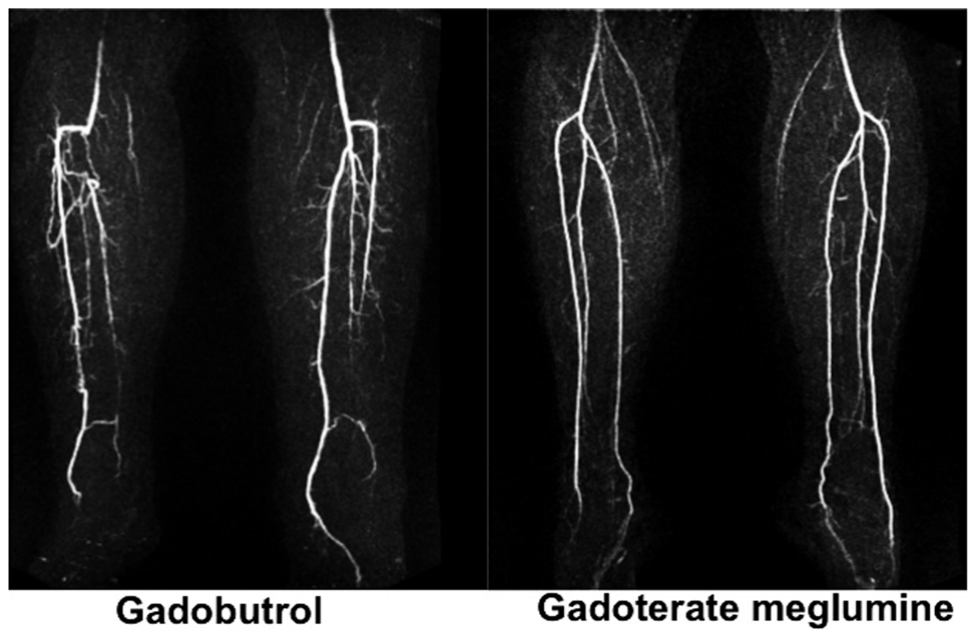
Higher SNR and CNR in time resolved MR-Angiography does not translate into improved image quality. Gadobutrol was utilized as the contrast agent in a 80 year old female with SNR/CNR values of 93/65 for the anterior tibial artery, 125/101 for the posterior tibial artery and 90/62 for the peroneal artery. Gadoterate meglumine administered in a 61 year old female resulted in SNR/CNR values of 60/21 for the anterior tibial artery, 55/21 for the posterior tibial artery and 52/19 for the peroneal artery. Image quality was rated good to excellent by both readers in both cases.

For reader 1, qualitative assessments were possible for the gadobutrol scans in 91% (87/96) of cases for the anterior tibial artery, 81% (78/96) of cases for the posterior tibial artery, and 90% (86/96) of cases for the peroneal artery. For the gadoterate meglumine scans, these respective assessments were possible in 89% (85/96), (89%) 85/96, and 93% (89/96) of cases. For reader 2, qualitative assessments were possible for the gadobutrol scans in 91% (87/96) of cases for the anterior tibial artery, 80% (77/96) of cases for the posterior tibial artery, and 90% (86/96) of cases for the peroneal artery. For the gadoterate meglumine scans these respective assessments were possible in 90% (85/96), 90% (85/96), and 92% (88/96) of cases. Image quality ratings ranged from values of 1–4 for each reader. Median ratings of image quality are provided in Table 3. Statistically significant differences in image quality were observed between gadobutrol and gadoterate meglumine in the peroneal arteries for both readers. Kappa values demonstrated excellent interobserver agreement for all qualitative assessments (Table 3). An example of individual SNR and CNR values as well as the pertinent image quality ratings by both readers is provided in figure 1.

**Table 3:** Image Quality.

		Median Rating		
Distribution	Reader	(Gadobutrol, Gd-DOTA)	P-values	Kappa**
Anterior Tibial Artery	Reader 1	4, 4	0.29	0.88
	Reader 2	4, 4	0.42	
Posterior Tibial Artery	Reader 1	4, 4	0.56	0.93
	Reader 2	4, 4	0.68	
Peroneal Artery	Reader 1	4, 4	0.006*	0.87
	Reader 2	4, 4	0.04*	

*Statistically significantly better image quality with gadobutrol.

**All Kappa values are statistically significant.

Gd-DOTA  =  gadoterate meglumine.

## Discussion

The results of the present study demonstrate superior SNR and CNR for equimolar doses of gadobutrol versus gadoterate meglumine for time-resolved MRA of the calves at 3 T. Our results demonstrate a significant difference in SNR and CNR measurements in all three vessel segments evaluated, with gadobutrol achieving SNR and CNR values up to 100% higher than gadoterate meglumine. However, this did not translate into the expected degree of improved subjective image quality as gadobutrol scans were only rated to have statistically significantly better image quality in one of three assessed vascular segments.

A previous study by Voth et al. showed equimolar doses of gadobutrol, also diluted to 0.5 M with saline, provided greater image quality for peripheral MRA in all vessel segments from the diaphragm to the calves versus gadoterate meglumine utilizing CTM-MRA. Both contrast agents were injected at a dose of 0.07 mmol/kg bw in this instance and at a constant injection rate of 1.5 ml/sec [17]. A recent study performed by Haneder et al. compared SNR, CNR, and image quality on an intraindividual basis in 14 patients who underwent static, CTM-MRA using either gadobutrol or gadoterate meglumine [18]. Haneder et al. found that while SNR and CNR were statistically significantly greater for gadobutrol, gadoterate meglumine images were rated as superior in terms of image quality and diagnostic confidence. In distinction to that work, our study included a larger number of patients and focused solely on time-resolved imaging of the calves. The value of TWIST-MRA relative to static MRA is primarily due to its stability in conditions of altered flow, conditions often present in higher stages of PAD [4]. Contrast agent comparisons for MR angiography applications often lead to controversial discussions focusing on study design and the fairness of comparisons between agents. Crucial considerations include injection dose, volume, and rate. The complexity of comparisons increases when comprehensive evaluations of contrast agents at non-equimolar formulations are performed. Considering these factors, the mixed conclusions of prior studies in this field are not surprising. Achenbach et al. found no significant differences in terms of image quality, diagnostic accuracy, signal intensity, SNR, and CNR between 0.5 M gadobenate dimeglumine and 1.0 M gadobutrol in 74 Patients at 1.5 T [19]. Gadobenate dimeglumine is known to bind temporarily to serum proteins such as albumin, which in consequence leads to an increased r^1^-relaxation rate relative to non-protein binding agents like gadobutrol [20].

Szucs-Farkas et al. concluded that gadobutrol did not show significant differences in regards to SNR, CNR or image quality in CE-MRA performed at 1.5 Tesla compared to gadoterate meglumine. Szucs-Farkas et al. did not dilute gadoterate meglumine, but did inject the agents at equimolar doses per bodyweight. The major drawback of that study was the slow injection rates of 0.4 and 0.8 ml/sec for gadobutrol and gadoterate meglumine, respectively. This may have resulted in insufficient bolus definition, particularly in the peripheral vasculature of the calves. The authors concluded that further investigation was warranted to determine whether a higher injection rate would translate into better image quality [21]. Comparison of results obtained at 3 and 1.5 Tesla is even more difficult due to differences in relaxivity of contrast agents at different field strengths [15] and different T1 relaxation times of the background tissue.

Our study utilized an equimolar dose of the contrast agents, with dilution of the 1 M gadobutrol 1∶1 with normal saline (NaCl), thus allowing for an equivalent concentration and similar bolus geometry compared to the 0.5 M gadoterate meglumine. Dilution allowed for injection of the same volume of contrast material for both injection protocols, minimizing the potential for technologist error. Thus, using the same technical parameters, gadobutrol performed significantly better than gadoterate meglumine in terms of objective image quality. However, our initital hypothesis that the higher relaxivity of gadoterate would translate into improved diagnostic image quality did not hold true. This is in agreement with prior work by Fink et al. and Haneder et al. [18], [22], who suggested that measurable differences in SNR and CNR are irrelevant for subjectively rated image quality.

Several limitations of our study warrant further discussion. Neither age, gender, nor PAD stage was matched; however, both groups contain similar numbers of male and female patients of similar mean ages. Contrast agent was already present in the vessel segments evaluated on pre-contrast scans due to the prior CTM-MRA, however this possible confounder was equivalently present in both groups. In addition, intra-individual comparisons could not be performed due to the retrospective study design. By definition, retrospective studies are limited, and therefore larger scale prospective studies are required to validate our results.

In conclusion, the significantly better enhancement as assessed by SNR and CNR provided by gadobutrol does not translate into improved image quality in an equimolar, low-dose, time-resolved MRA protocol.
